# (5*Z*)-5-(2-Hydroxy­benzyl­idene)-3-phenyl-2-thioxo-1,3-thia­zolidin-4-one

**DOI:** 10.1107/S1600536809044286

**Published:** 2009-10-28

**Authors:** Durre Shahwar, M. Nawaz Tahir, Muhammad Asam Raza, Bushra Iqbal

**Affiliations:** a Department of Chemistry, Government College University, Lahore, Pakistan; b Department of Physics, University of Sargodha, Sargodha, Pakistan

## Abstract

In the title compound, C_16_H_11_NO_2_S_2_, the dihedral angles between the heterocyclic ring and the phenyl and anilinic benzene rings are 9.68 (13) and 79.26 (6)°, respectively, and an intra­molecular C—H⋯S inter­action occurs. In the crystal, inversion dimers linked by pairs of O—H⋯O hydrogen bonds occur, leading to *R*_2_^2^(10) loops, and C—H⋯O and weak C—H⋯π inter­actions further consolidate the packing.

## Related literature

For related structures, see: Linden *et al.* (1999 ▶); Shahwar *et al.* (2009*a* ▶, 2009*b* ▶). For graph-set theory, see: Bernstein *et al.* (1995 ▶).
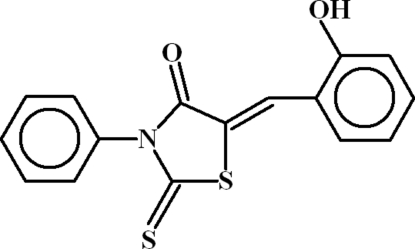


## Experimental

### 

#### Crystal data

C_16_H_11_NO_2_S_2_
*M* *_r_* = 313.40
Monoclinic, 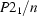

*a* = 11.6553 (7) Å
*b* = 7.3424 (4) Å
*c* = 16.8256 (10) Å
β = 95.131 (2)°
*V* = 1434.13 (14) Å^3^
*Z* = 4
Mo *K*α radiation
μ = 0.37 mm^−1^
*T* = 296 K
0.26 × 0.18 × 0.17 mm

#### Data collection

Bruker Kappa APEXII CCD diffractometer
Absorption correction: multi-scan (*SADABS*; Bruker, 2005 ▶) *T*_min_ = 0.924, *T*_max_ = 0.937
15580 measured reflections
3481 independent reflections
2194 reflections with *I* > 2σ(*I*)
*R*_int_ = 0.048

#### Refinement

*R*[*F* ^2^ > 2σ(*F* ^2^)] = 0.041
*wR*(*F* ^2^) = 0.102
*S* = 1.01
3481 reflections
191 parameters
H-atom parameters constrained
Δρ_max_ = 0.26 e Å^−3^
Δρ_min_ = −0.27 e Å^−3^

### 

Data collection: *APEX2* (Bruker, 2007 ▶); cell refinement: *SAINT* (Bruker, 2007 ▶); data reduction: *SAINT*; program(s) used to solve structure: *SHELXS97* (Sheldrick, 2008 ▶); program(s) used to refine structure: *SHELXL97* (Sheldrick, 2008 ▶); molecular graphics: *ORTEP-3* (Farrugia, 1997 ▶) and *PLATON* (Spek, 2009 ▶); software used to prepare material for publication: *WinGX* (Farrugia, 1999 ▶) and *PLATON*.

## Supplementary Material

Crystal structure: contains datablocks global, I. DOI: 10.1107/S1600536809044286/hb5180sup1.cif

Structure factors: contains datablocks I. DOI: 10.1107/S1600536809044286/hb5180Isup2.hkl

Additional supplementary materials:  crystallographic information; 3D view; checkCIF report

## Figures and Tables

**Table 1:** Hydrogen-bond geometry (Å, °)

*D*—H⋯*A*	*D*—H	H⋯*A*	*D*⋯*A*	*D*—H⋯*A*
C16—H16⋯S1	0.93	2.50	3.213 (2)	133
O2—H2*A*⋯O1^i^	0.82	1.97	2.767 (2)	163
C10—H10⋯O2^i^	0.93	2.49	3.375 (3)	160
C2—H2⋯CgB^ii^	0.93	2.91	3.774 (2)	155
C14—H14⋯CgB^iii^	0.93	2.91	3.515 (2)	124

Symmetry codes: (i) 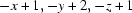; (ii) 
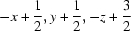
; (iii) 
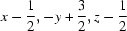
. *CgB* is the centroid of the C1–C6 ring.

**Fig. 1.**
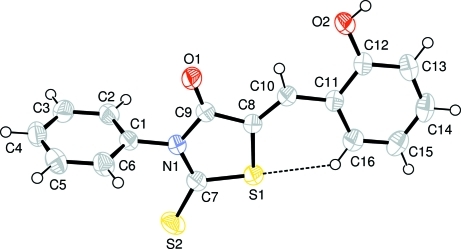
View of (I) with displacement ellipsoids drawn at the 50% probability level. The dotted line represents the intramolecular H-bond.

**Fig. 2.**
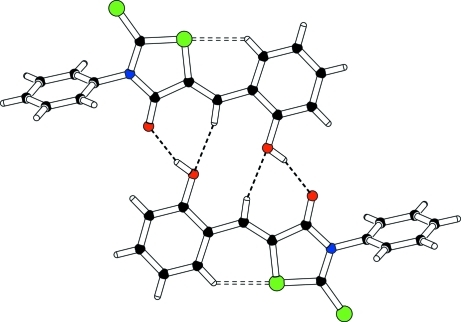
The partial packing of (I), which shows that molecules form inversion dimers.

## supplementary crystallographic information

### Comment

We have recently reported the crystal structure of (II) (5*Z*)-5-(2-Hydroxybenzylidene)-2-thioxo-1,3-thiazolidin-4- one methanol hemisolvate (Shahwar *et al.*, 2009*a*) and (III) (5*E*)-5-(4-Hydroxy-3-methoxybenzylidene)-2-thioxo-1,3-thiazolidin- 4-one methanol monosolvate (Shahwar *et al.*, 2009*b*) which contain the rhodanine. In continuation of synthesizing various derivatives of rhodanine, the title compound (I, Fig. 1), is being reported. The crystal structure of (IV) 3-Phenyl-5-(phenylmethylidene)-2-thioxo-1,3-thiazolidin-4-one (Linden *et al.*, 1999) has also been published. Title compound (I) differs from (IV) due to attachement of hydroxy group with benzylidene.

In (I) the heterocyclic ring A (N1/C7/S1/C8/C9), two benzene rings B (C1—C6) and C (C11–C16) are planar with maximum r. m. s. deviations of 0.0145, 0.0038 and 0.0070 Å respectively, from the respective mean square planes. The dihedral angles between A/B, A/C and B/C are 79.26 (6), 9.68 (13) and 69.62 (6)°, respectively. The intramolecular H-bondings of C—H···S (Table 1, Fig. 1) form twisted S(6) ring motif (Bernstein *et al.*, 1995). The molecules of (I) are stabilized in the form of dimers due to intermolecular H-bondings (Table 1, Fig. 2) froming *R*_2_^2^(7) and *R*_2_^2^(10) ring motifs. The C–H···π interactions (Table 1) also play role in stabilizing the molecules.

### Experimental

3-Phenyl-2-thioxo-1,3-thiazolidin-4-one (0.419 g, 0.2 mol), 2-Hydroxybenzaldehyde (0.244 g, 0.2 mol) and K_2_CO_3_ (0.553 g, 0.4 mol) were dissolved in 10 ml distilled water at room temperature. The stirring was continued for 24 h and reaction was monitored by TLC. The precipitates were formed during neutalization of the reaction mixture with 5% HCl. The precipitates were filtered off and washed with saturated solution of NaCl. The crude material obtained was recrystalized in ethyl acetate to affoard orange yellow prisms of (I).

### Refinement

The H-atoms were positioned geometrically (O–H = 0.82 Å, C–H = 0.93 Å) and refined as riding with *U*_iso_(H) = 1.2*U*_eq_(C) or 1.5*U*_eq_(O).

### Crystal data

**Table:**

C_16_H_11_NO_2_S_2_	*F*(000) = 648
*M**_r_* = 313.40	*D*_x_ = 1.452 Mg m^−^^3^
Monoclinic, *P*2_1_/*n*	Mo *K*α radiation, λ = 0.71073 Å
Hall symbol: -P 2yn	Cell parameters from 3481 reflections
*a* = 11.6553 (7) Å	θ = 2.0–28.0°
*b* = 7.3424 (4) Å	µ = 0.37 mm^−^^1^
*c* = 16.8256 (10) Å	*T* = 296 K
β = 95.131 (2)°	Prisms, orange yellow
*V* = 1434.13 (14) Å^3^	0.26 × 0.18 × 0.17 mm
*Z* = 4	

### Data collection

**Table:**

Bruker Kappa APEXII CCD diffractometer	3481 independent reflections
Radiation source: fine-focus sealed tube	2194 reflections with *I* > 2σ(*I*)
graphite	*R*_int_ = 0.048
Detector resolution: 7.40 pixels mm^-1^	θ_max_ = 28.0°, θ_min_ = 2.0°
ω scans	*h* = −15→14
Absorption correction: multi-scan (*SADABS*; Bruker, 2005)	*k* = −9→9
*T*_min_ = 0.924, *T*_max_ = 0.937	*l* = −22→22
15580 measured reflections	

### Refinement

**Table:**

Refinement on *F*^2^	Primary atom site location: structure-invariant direct methods
Least-squares matrix: full	Secondary atom site location: difference Fourier map
*R*[*F*^2^ > 2σ(*F*^2^)] = 0.041	Hydrogen site location: inferred from neighbouring sites
*wR*(*F*^2^) = 0.102	H-atom parameters constrained
*S* = 1.01	*w* = 1/[σ^2^(*F*_o_^2^) + (0.0375*P*)^2^ + 0.326*P*] where *P* = (*F*_o_^2^ + 2*F*_c_^2^)/3
3481 reflections	(Δ/σ)_max_ < 0.001
191 parameters	Δρ_max_ = 0.26 e Å^−^^3^
0 restraints	Δρ_min_ = −0.27 e Å^−^^3^

### Special details

**Table:**

Geometry. Bond distances, angles *etc*. have been calculated using the rounded fractional coordinates. All su's are estimated from the variances of the (full) variance-covariance matrix. The cell e.s.d.'s are taken into account in the estimation of distances, angles and torsion angles
Refinement. Refinement of *F*^2^ against ALL reflections. The weighted *R*-factor *wR* and goodness of fit *S* are based on *F*^2^, conventional *R*-factors *R* are based on *F*, with *F* set to zero for negative *F*^2^. The threshold expression of *F*^2^ > σ(*F*^2^) is used only for calculating *R*-factors(gt) *etc*. and is not relevant to the choice of reflections for refinement. *R*-factors based on *F*^2^ are statistically about twice as large as those based on *F*, and *R*- factors based on ALL data will be even larger.

### Fractional atomic coordinates and isotropic or equivalent isotropic displacement parameters (Å^2^)

**Table:**

	*x*	*y*	*z*	*U*_iso_*/*U*_eq_	
S1	0.16936 (5)	0.50419 (7)	0.48587 (3)	0.0463 (2)	
S2	0.12725 (6)	0.16709 (8)	0.57270 (4)	0.0591 (2)	
O1	0.45774 (15)	0.5967 (2)	0.60654 (9)	0.0572 (6)	
O2	0.40155 (14)	1.1358 (2)	0.44795 (9)	0.0515 (6)	
N1	0.30920 (15)	0.3922 (2)	0.60195 (9)	0.0376 (6)	
C1	0.35642 (18)	0.2866 (3)	0.66982 (12)	0.0370 (7)	
C2	0.3410 (2)	0.3461 (3)	0.74551 (12)	0.0432 (7)	
C3	0.3835 (2)	0.2416 (3)	0.81002 (12)	0.0477 (8)	
C4	0.4398 (2)	0.0815 (3)	0.79786 (14)	0.0497 (8)	
C5	0.4550 (2)	0.0235 (3)	0.72188 (14)	0.0524 (9)	
C6	0.4139 (2)	0.1273 (3)	0.65678 (13)	0.0466 (8)	
C7	0.20684 (19)	0.3439 (3)	0.55947 (12)	0.0399 (7)	
C8	0.29274 (19)	0.6347 (3)	0.51013 (11)	0.0385 (7)	
C9	0.3643 (2)	0.5470 (3)	0.57636 (12)	0.0401 (7)	
C10	0.32304 (19)	0.7938 (3)	0.47846 (12)	0.0418 (7)	
C11	0.26629 (19)	0.9014 (3)	0.41462 (11)	0.0379 (7)	
C12	0.31041 (19)	1.0743 (3)	0.39934 (12)	0.0389 (7)	
C13	0.2611 (2)	1.1796 (3)	0.33719 (13)	0.0494 (8)	
C14	0.1673 (2)	1.1153 (3)	0.28976 (13)	0.0540 (9)	
C15	0.1207 (2)	0.9486 (3)	0.30463 (13)	0.0533 (9)	
C16	0.1702 (2)	0.8438 (3)	0.36550 (13)	0.0478 (8)	
H2	0.30268	0.45483	0.75331	0.0518*	
H2A	0.43058	1.22336	0.42710	0.0772*	
H3	0.37382	0.28003	0.86166	0.0572*	
H4	0.46807	0.01166	0.84139	0.0596*	
H5	0.49284	−0.08572	0.71418	0.0629*	
H6	0.42501	0.08995	0.60520	0.0559*	
H10	0.39181	0.84263	0.50141	0.0502*	
H13	0.29121	1.29390	0.32743	0.0593*	
H14	0.13538	1.18544	0.24737	0.0648*	
H15	0.05592	0.90715	0.27356	0.0640*	
H16	0.13881	0.72999	0.37452	0.0574*	

### Atomic displacement parameters (Å^2^)

**Table:**

	*U*^11^	*U*^22^	*U*^33^	*U*^12^	*U*^13^	*U*^23^
S1	0.0458 (4)	0.0443 (3)	0.0466 (3)	−0.0085 (3)	−0.0083 (2)	0.0067 (2)
S2	0.0556 (4)	0.0454 (3)	0.0756 (4)	−0.0154 (3)	0.0014 (3)	0.0099 (3)
O1	0.0538 (11)	0.0551 (10)	0.0582 (10)	−0.0197 (8)	−0.0194 (8)	0.0209 (8)
O2	0.0534 (11)	0.0476 (9)	0.0518 (9)	−0.0149 (8)	−0.0042 (8)	0.0148 (7)
N1	0.0414 (11)	0.0340 (9)	0.0369 (9)	−0.0023 (8)	0.0001 (8)	0.0047 (7)
C1	0.0387 (13)	0.0322 (10)	0.0397 (11)	−0.0023 (9)	0.0020 (9)	0.0042 (8)
C2	0.0475 (14)	0.0371 (11)	0.0449 (12)	0.0020 (10)	0.0036 (10)	0.0007 (9)
C3	0.0533 (16)	0.0514 (13)	0.0380 (12)	−0.0027 (12)	0.0027 (10)	0.0026 (10)
C4	0.0480 (15)	0.0486 (13)	0.0514 (14)	−0.0004 (11)	−0.0012 (11)	0.0169 (11)
C5	0.0556 (17)	0.0388 (12)	0.0631 (15)	0.0100 (11)	0.0071 (12)	0.0076 (10)
C6	0.0573 (16)	0.0412 (12)	0.0424 (12)	0.0060 (10)	0.0100 (11)	0.0015 (9)
C7	0.0418 (14)	0.0364 (11)	0.0418 (11)	−0.0014 (10)	0.0053 (9)	0.0003 (9)
C8	0.0427 (13)	0.0362 (11)	0.0357 (11)	−0.0038 (9)	−0.0014 (9)	0.0027 (8)
C9	0.0463 (15)	0.0355 (11)	0.0378 (11)	−0.0051 (9)	0.0004 (10)	0.0036 (8)
C10	0.0428 (14)	0.0422 (12)	0.0391 (11)	−0.0053 (10)	−0.0038 (9)	0.0042 (9)
C11	0.0391 (13)	0.0406 (11)	0.0337 (11)	0.0012 (10)	0.0025 (9)	0.0042 (8)
C12	0.0390 (13)	0.0428 (11)	0.0350 (11)	0.0039 (10)	0.0040 (9)	0.0054 (9)
C13	0.0561 (16)	0.0453 (12)	0.0478 (13)	0.0090 (11)	0.0097 (12)	0.0138 (10)
C14	0.0571 (17)	0.0631 (16)	0.0413 (13)	0.0211 (13)	0.0025 (12)	0.0134 (11)
C15	0.0471 (16)	0.0672 (16)	0.0438 (13)	0.0075 (12)	−0.0066 (11)	0.0018 (11)
C16	0.0488 (15)	0.0485 (13)	0.0451 (12)	−0.0022 (11)	−0.0019 (10)	0.0041 (10)

### Geometric parameters (Å, °)

**Table:**

S1—C7	1.736 (2)	C10—C11	1.445 (3)
S1—C8	1.746 (2)	C11—C16	1.397 (3)
S2—C7	1.623 (2)	C11—C12	1.402 (3)
O1—C9	1.216 (3)	C12—C13	1.384 (3)
O2—C12	1.359 (3)	C13—C14	1.378 (3)
O2—H2A	0.8200	C14—C15	1.371 (3)
N1—C7	1.381 (3)	C15—C16	1.367 (3)
N1—C9	1.393 (3)	C2—H2	0.9300
N1—C1	1.448 (3)	C3—H3	0.9300
C1—C6	1.375 (3)	C4—H4	0.9300
C1—C2	1.373 (3)	C5—H5	0.9300
C2—C3	1.384 (3)	C6—H6	0.9300
C3—C4	1.371 (3)	C10—H10	0.9300
C4—C5	1.374 (3)	C13—H13	0.9300
C5—C6	1.384 (3)	C14—H14	0.9300
C8—C9	1.479 (3)	C15—H15	0.9300
C8—C10	1.344 (3)	C16—H16	0.9300
			
C7—S1—C8	93.20 (10)	C11—C12—C13	120.7 (2)
C12—O2—H2A	109.00	O2—C12—C11	118.06 (18)
C1—N1—C9	121.85 (17)	C12—C13—C14	120.1 (2)
C7—N1—C9	116.78 (17)	C13—C14—C15	120.5 (2)
C1—N1—C7	121.37 (16)	C14—C15—C16	119.5 (2)
N1—C1—C6	119.07 (18)	C11—C16—C15	122.4 (2)
C2—C1—C6	121.6 (2)	C1—C2—H2	121.00
N1—C1—C2	119.28 (19)	C3—C2—H2	121.00
C1—C2—C3	118.9 (2)	C2—C3—H3	120.00
C2—C3—C4	120.1 (2)	C4—C3—H3	120.00
C3—C4—C5	120.5 (2)	C3—C4—H4	120.00
C4—C5—C6	120.1 (2)	C5—C4—H4	120.00
C1—C6—C5	118.8 (2)	C4—C5—H5	120.00
S1—C7—S2	122.00 (13)	C6—C5—H5	120.00
S1—C7—N1	110.23 (15)	C1—C6—H6	121.00
S2—C7—N1	127.77 (16)	C5—C6—H6	121.00
S1—C8—C9	109.54 (15)	C8—C10—H10	115.00
S1—C8—C10	128.64 (17)	C11—C10—H10	115.00
C9—C8—C10	121.8 (2)	C12—C13—H13	120.00
O1—C9—C8	127.4 (2)	C14—C13—H13	120.00
N1—C9—C8	110.11 (18)	C13—C14—H14	120.00
O1—C9—N1	122.45 (19)	C15—C14—H14	120.00
C8—C10—C11	130.6 (2)	C14—C15—H15	120.00
C10—C11—C16	124.3 (2)	C16—C15—H15	120.00
C12—C11—C16	116.97 (19)	C11—C16—H16	119.00
C10—C11—C12	118.71 (19)	C15—C16—H16	119.00
O2—C12—C13	121.3 (2)		
			
C8—S1—C7—S2	−179.12 (15)	C3—C4—C5—C6	0.3 (4)
C8—S1—C7—N1	1.29 (16)	C4—C5—C6—C1	−1.1 (3)
C7—S1—C8—C9	0.95 (16)	S1—C8—C9—O1	177.26 (19)
C7—S1—C8—C10	−177.2 (2)	S1—C8—C9—N1	−2.9 (2)
C7—N1—C1—C2	−99.9 (2)	C10—C8—C9—O1	−4.5 (4)
C7—N1—C1—C6	78.9 (3)	C10—C8—C9—N1	175.33 (19)
C9—N1—C1—C2	80.0 (3)	S1—C8—C10—C11	−1.9 (4)
C9—N1—C1—C6	−101.2 (2)	C9—C8—C10—C11	−179.8 (2)
C1—N1—C7—S1	176.46 (14)	C8—C10—C11—C12	172.8 (2)
C1—N1—C7—S2	−3.1 (3)	C8—C10—C11—C16	−8.2 (4)
C9—N1—C7—S1	−3.5 (2)	C10—C11—C12—O2	−3.0 (3)
C9—N1—C7—S2	176.98 (17)	C10—C11—C12—C13	177.9 (2)
C1—N1—C9—O1	4.1 (3)	C16—C11—C12—O2	177.90 (19)
C1—N1—C9—C8	−175.75 (17)	C16—C11—C12—C13	−1.2 (3)
C7—N1—C9—O1	−176.0 (2)	C10—C11—C16—C15	−178.6 (2)
C7—N1—C9—C8	4.2 (2)	C12—C11—C16—C15	0.5 (3)
N1—C1—C2—C3	178.1 (2)	O2—C12—C13—C14	−178.7 (2)
C6—C1—C2—C3	−0.7 (3)	C11—C12—C13—C14	0.3 (3)
N1—C1—C6—C5	−177.6 (2)	C12—C13—C14—C15	1.3 (3)
C2—C1—C6—C5	1.3 (3)	C13—C14—C15—C16	−2.0 (3)
C1—C2—C3—C4	−0.1 (3)	C14—C15—C16—C11	1.1 (3)
C2—C3—C4—C5	0.2 (4)		

### Hydrogen-bond geometry (Å, °)

**Table:**

*D*—H···*A*	*D*—H	H···*A*	*D*···*A*	*D*—H···*A*
C16—H16···S1	0.93	2.50	3.213 (2)	133
O2—H2A···O1^i^	0.82	1.97	2.767 (2)	163
C10—H10···O2^i^	0.93	2.49	3.375 (3)	160
C2—H2···CgB^ii^	0.93	2.91	3.774 (2)	155
C14—H14···CgB^iii^	0.93	2.91	3.515 (2)	124

Symmetry codes: (i) −*x*+1, −*y*+2, −*z*+1; (ii) −*x*+1/2, *y*+1/2, −*z*+3/2; (iii) *x*−1/2, −*y*+3/2, *z*−1/2.
